# Addressing Disciplinary Misconceptions: Mentorship Programs and Student-Led Surgical Societies. Comment on “Perception of Plastic Surgery and the Role of Media Among Medical Students: Cross-Sectional Study”

**DOI:** 10.2196/17237

**Published:** 2021-03-04

**Authors:** Grace Olivia Jane Poole, Naomi Melamed, Ella Hubbard

**Affiliations:** 1 St George's University of London London United Kingdom

**Keywords:** medical education, plastic surgery, students

We would like to thank Mortada et al [1] for their recent article, highlighting the misconceptions of plastic surgery among medical students. Upon reading the article, we were surprised by the lack of awareness reported among both female and male students.

As the authors highlighted, medical students generally possess a poor understanding of what plastic surgery entails. These results are not unique to Saudi Arabia, with analysis of medical schools in both the United Kingdom and Canada indicating that students are unaware of the full breadth of operations plastic surgeons perform, including the management of pressure ulcers, cleft palate, and carpal tunnel syndrome [2,3]. Given the competitive nature of plastic surgery globally, it is important that students begin to construct competitive portfolios early [4]. However, if students are unaware of the full range and utility of plastic surgery, it is questionable how they may be expected to do so. Moreover, only 26% of qualified doctors would refer neuronal injuries of the hand to plastic surgeons [5]. This highlights a fundamental gap in the understanding of plastic surgery among both students and qualified practitioners.

We recognize that many students engage with medical television programs and agree that accurate multimedia representation of plastic surgery is important. However, we disagree that television represents a viable tool to promote student awareness of the full range, complexity, and utility of plastic surgery. In fact, there is evidence that television representation of plastic surgery may actively contribute to a skewed understanding of the specialty among medical students [3]. Similarly, content on social media is likely to reinforce existing misconceptions around the work of plastic surgeons, with research suggesting that surgeons with a cosmetic focus to their practice are more likely to utilize social media [6]. Furthermore, content on social media platforms is unregulated, and the reliability of information cannot be guaranteed.

We propose that there are more effective strategies to promote student understanding of plastic surgery. Exposure to plastic surgery during medical school has been shown to increase matriculation into the specialty; yet only 29.4% of students report such exposure [2,7]. One method shown to effectively overcome this gap in the formal curriculum is extracurricular mentorship programs. Within these programs, students can shadow plastic surgeons in their clinical environment, gaining insights into the realities of the specialty [8]. In addition, there is a clear role to be played by proactive interaction with student-led surgical societies. Student-organized career sessions allow trainees to deliver accurate information on the variety, demands, and rewards of a career in plastic surgery. Mentorship programs and society-organized careers sessions both enable students to develop a realistic understanding of plastic surgery and offer the opportunity for students to develop competitive portfolios.

Overall, we agree with Mortada et al [1] that students possess an inadequate understanding of plastic surgery. However, we propose that more effective strategies to overcome this would include mentorship programs and student-led surgical societies, rather than potentially skewed representations of the specialty on television and social media.

## References

[1] Mortada Hatan Hisham, Alqahtani Yara Aayed, Seraj Hadeel Zakaria, Albishi Wahbi Khalid, Aljaaly Hattan A Perception of Plastic Surgery and the Role of Media Among Medical Students: Cross-Sectional Study. Interact J Med Res.

[2] Farid M, Vaughan R, Thomas S (2017). Plastic Surgery Inclusion in the Undergraduate Medical Curriculum: Perception, Challenges, and Career Choice-A Comparative Study. Plast Surg Int.

[3] Fraser S, Al Youha S, Rasmussen P J, Williams J G (2017). Medical Student Perception of Plastic Surgery and the Impact of Mainstream Media. Plast Surg (Oakv).

[4] Morzycki A, Bezuhly Michael, Williams Jason G (2018). How Competitive Is Plastic Surgery? An Analysis of the Canadian and American Residency Match. Plast Surg (Oakv).

[5] Panse N, Panse Smita, Kulkarni Priya, Dhongde Rajendra, Sahasrabudhe Parag (2012). Awareness and Perception of Plastic Surgery among Healthcare Professionals in Pune, India: Do They Really Know What We Do?. Plast Surg Int.

[6] Vardanian Andrew J, Kusnezov Nicholas, Im Daniel D, Lee James C, Jarrahy Reza (2013). Social media use and impact on plastic surgery practice. Plast Reconstr Surg.

[7] Agarwal Jayant P, Mendenhall Shaun D, Moran Linh A, Hopkins Paul N (2013). Medical student perceptions of the scope of plastic and reconstructive surgery. Ann Plast Surg.

[8] Barker Jenny C, Rendon Juan, Janis Jeffrey E (2016). Medical Student Mentorship in Plastic Surgery: The Mentee's Perspective. Plast Reconstr Surg.

